# Association of aortic diameters and mortality in the general population—an MRI-based study

**DOI:** 10.1007/s00330-024-10965-4

**Published:** 2024-07-18

**Authors:** Birger Mensel, Andreas H. Mahnken, Erhard Kaiser, Henry Völzke, Marcus Dörr, Stephan B. Felix, Till Ittermann, Wolfgang Lieb, Roberto Lorbeer

**Affiliations:** 1 Department of Diagnostic & Interventional Radiology, Philipps-University Marburg, Marburg, Germany; 2 Department of Diagnostic and Interventional Radiology and Neuroradiology, Central Hospital Bad Berka, Bad Berka, Germany; 3 Institute for Community Medicine, University Medicine Greifswald, Greifswald, Germany; 4 Department of Internal Medicine B, University Medicine Greifswald, Greifswald, Germany; 5 German Centre for Cardiovascular Research (DZHK e.V.), Partner Site Greifswald, Greifswald, Germany; 6 Institute of Epidemiology, Kiel University, Kiel, Germany; 7 Department of Radiology, University Hospital LMU Munich, Munich, Germany; 8 German Centre for Cardiovascular Research (DZHK e.V.), Partner Site Munich Heart Alliance, Munich, Germany

**Keywords:** Thoracic aorta, Abdominal aorta, Magnetic resonance imaging, Cardiovascular diseases

## Abstract

**Background:**

Increased diameters of the aorta are associated with increased mortality risk. In the present analyses, we assessed whether aortic diameters are associated with cardiovascular and all-cause mortality in community-dwelling individuals free of known cardiovascular disease (CVD).

**Methods:**

MRI-derived vascular parameters of the thoracic and abdominal aorta from 2668 participants (median age = 53 years; 51.1% women) of the population-based SHIP-START-2 and SHIP-TREND-0 cohorts without CVD were analyzed. Age- and sex-adjusted, as well as multivariable-adjusted Cox-proportional hazard models, were used to estimate associations of diameters of six different aortic segments to mortality.

**Results:**

Over a median follow-up time of 10.6 years (IQR: 8.7; 12.4), a total of 188 participants (126 men and 62 women) died, of which 38 deaths were due to CVD. In unadjusted models, mortality rates were higher in participants with aortic diameters above the median compared to below the median for all investigated aortic sections (all log-rank *p* < 0.001). In multivariable-adjusted models, the diameters of the ascending thoracic aorta (HR = 1.34 95% CI: 1.04; 1.72, *p* = 0.022) and of the infrarenal aorta (HR = 3.75 95% CI: 1.06; 13.3, *p* = 0.040), modeled continuously, were associated with greater cardiovascular mortality. The diameter of the subphrenic aorta was associated with higher cardiovascular mortality only in the age and sex-adjusted model (HR = 3.65 95% CI: 1.01; 13.3, *p* = 0.049). None of the investigated aortic segments were associated with all-cause mortality.

**Conclusion:**

Non-indexed diameters of the ascending thoracic and infrarenal aorta were associated with higher cardiovascular mortality but not with all-cause mortality in a population sample free of clinically overt CVD at baseline.

**Clinical relevance statement:**

Increased aortic diameter is associated with cardiovascular mortality and can help to identify high-risk patients.

**Key Points:**

- *Increased aortic diameter is associated with mortality*.
- *Non-indexed diameters of the ascending and infrarenal aorta are associated with cardiovascular mortality but not all-cause mortality*.
- *Aortic diameter measurements support the estimate of cardiovascular mortality*.

## Introduction

The aortic diameter is associated beside sex, age, and body surface area (BSA) with traditional risk factors of cardiovascular disease (CVD) such as smoking, blood pressure, and dyslipidemia [1, 2]. It is well known that increased aortic diameter is correlated with the risk of life-threatening aortic wall diseases such as dissection and aneurysm formation [3, 4]. However, there is also some evidence that even a subclinical increase in aortic diameter at various anatomic locations is associated with adverse CVD events and mortality. A recent study showed that larger BMI-indexed ascending and descending aortic diameters are associated with a greater risk of cardiovascular mortality in women and all-cause mortality in both sexes [5]. However, a previous study found, that increased infrarenal abdominal aorta and lower abdominal aorta were associated with an increased risk of CVD events in women and men (including cardiovascular death). In contrast, increased diameters of the ascending and descending thoracic aorta were not significantly associated with CVD events [6].

The pathophysiology leading to aortic dilation is complex and not fully understood, but the final common path of all mechanisms is aortic wall degeneration with a subsequent increase in diameter [7, 8]. The aortic wall at various anatomic locations is affected to a different extent by aortic wall stressors, leading to different degrees of diameter increase [6, 9].

Therefore, the aim of this study was to assess if thoracic and abdominal aortic segments were associated with cardiovascular and all-cause mortality in a large population free of CVD at baseline.

## Methods

### Sample

The studied population was part of the Study of Health in Pomerania (SHIP). The study was approved by the Ethics Committee of the University of Greifswald and complies with the Declaration of Helsinki. All participants provided written informed consent.

SHIP is a prospective population-based project consisting of several independent cohorts established in the northeast area of Germany [10]. Between 2008 and 2012, the second examination cycle of the SHIP-START cohort (*N* = 2332) and the first examination cycle of the SHIP-TREND cohort (*N* = 4420) were conducted.

**Fig. 1:**
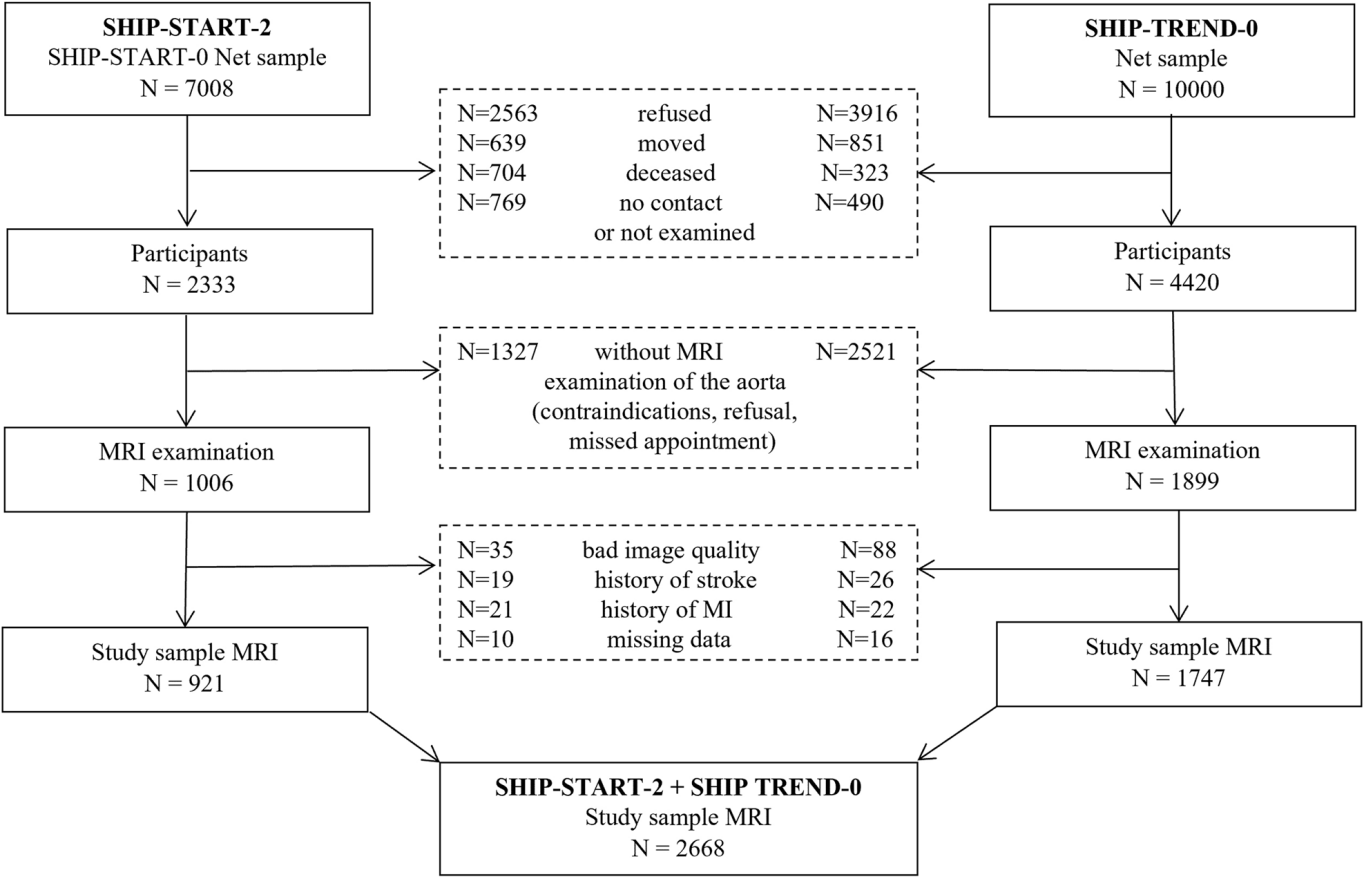
Flow-chart of the study sample drawn from the population-based cohorts SHIP-START-2 and SHIP-TREND-0

At these examination cycles, a total of 2905 participants received a cardiovascular MRI between 2008 and 2012, including imaging of the aorta. We excluded individuals with insufficient image quality (*N* = 123), persons with a history of stroke (*N* = 45), myocardial infarction (*N* = 43), and missing data for established cardiovascular risk factors (*N* = 26). The final analytical sample comprised 2668 Caucasian subjects (1336 women), aged 21–88 years (Fig. 1).

### Clinical examination and assessment of covariates

Participants were examined at the SHIP study center by trained personnel. Information on age and sex was provided by the population registries. In standardized personal interviews, information was collected on smoking status (categorized as never, ex-smoker, and current smoker), history of stroke, myocardial infarction, and diabetes. For the definition of current diabetes, a self-reported physician’s diagnosis or a glycated hemoglobin (HbA1c) value ≥ 6.5% was used.

Waist circumference was measured horizontally with an inelastic tape in the middle between the lower rib margin and the iliac crest. Body mass index (BMI) was calculated as weight (kg) divided by the square of height (m^2^). BSA was calculated according to the Du Bois formula: (BSA = 0.007184 × (height in cm)^0.725^ × (weight in kg)^0.425^ [11].

Systolic and diastolic blood pressure were measured three times at the right arm of seated participants using a digital blood pressure monitor (HEM-705CP, Omron Corporation) after a 5-min rest period, with each reading being followed by a further rest period of 3 min. The mean of the second and third measurements was calculated and used for present analyses.

Blood samples were drawn from the cubital vein in the supine position. The samples were taken between 07.00 a.m. and 04.00 p.m. and were analyzed immediately for standard laboratory parameters. Serum HbA1c levels were measured by high-performance liquid chromatography (Bio-Rad DiamatTM Analyzer, ClinRep® kit, RECIPE Chemicals + Instruments GmbH). Low-and high-density lipoprotein cholesterol (LDL-C and HDL-C), as well as triglyceride (TG) levels, were determined enzymatically using reagents from Dade Behring (Dimension VistaTM System, Flex® reagent cartridge, Dade Behring).

### MRI-derived aortic diameters

MRI was performed on a 1.5-T MRI scanner (Magnetom Avanto; Siemens Healthcare) using integrated coil elements and phased-array surface coils. Aortic diameters were measured on plain axial 3D T1-weighted volumetric interpolated breath-hold examination images.

The outer diameters (wall to wall) of six predefined aortic segments (three thoracic and three abdominal aortic segments) were measured: the ascending and descending aorta (level of the pulmonary trunk), the aortic arch (proximal to the origin of the left subclavian artery), the subdiaphragmatic aorta (level of the aortic hiatus), and the supra- and infrarenal aorta (1 cm above/below the right renal artery origin. Diameters were measured on plain axial MRI- slices. Further technical details of the aortic diameter measurements are described in detail elsewhere [1, 12].

### Mortality follow-up

Vital status information of study participants was collected regularly from population registries. Participants were censored at death or loss to follow-up. The number of months between baseline examination and censoring was used as the duration of follow-up. Death certificates were requested from the local health authorities and were coded by a certified nosologist according to the International Classification of Diseases, 10th revision (ICD-10). CVD was defined as ICD-10 codes I00 to I99 or R96. Two internists independently validated the underlying cause of death and performed a joint reading together with a third internist in cases of disagreement.

### Statistical analyses

Baseline characteristics of the study sample were summarized by median and interquartile range (IQR) for continuous data and by number and percentage for categorical data. The different aortic parameters that were used as exposure variables in our hazard models, were modeled as continuous traits in our primary analyses and were dichotomized at the median in further analyses.

Overall and cardiovascular survival curves were calculated by Kaplan–Meier analysis and compared by log-rank test between individuals above vs equal or below the median for each aortic segment diameter. Cox-proportional hazard models were used to estimate hazard ratios (HR) with a 95% confidence interval (CI) for overall and cardiovascular mortality associated with the aortic diameters in 6 aortic segments (each segment considered separately). The aortic diameters were modeled as continuous traits (HR are provided per 1-unit increment of aortic diameter). We adjusted for age and sex (Model 1) and for age, sex, smoking status, BMI, HbA1c, HDL-C, LDL-C, and TG (Model 2). These parameters were selected as confounders because they were associated with aortic parameters in a previous study [7]. We didn’t adjust the models for blood pressure since it is regarded as a mediator for the association between aortic parameters and mortality. The Cox-proportional hazards assumption was checked by the parallelism of survival curves of log-log plots and by Schoenfeld’s residuals.

The improvement in predictive accuracy by adding aortic diameters to a model based on traditional risk factors including age, sex, smoking status, BMI, systolic blood pressure, diastolic blood pressure, HbA1c, HDL-C, LDL-C, and TG was evaluated by Harrell’s C-index and Likelihood-ratio test.

In sensitivity analyses, non-linear associations and effect modifications were tested by multivariable regression spline models and by the Stata module “MFPIGEN”, respectively. In secondary analyses, parameters of aortic diameters were indexed by BSA and BMI. Multivariable models were adjusted for “cohort” to exclude potential unobserved methodical differences in further sub-analyses. A value of *p* < 0.05 was considered statistically significant. In addition, we used a Bonferroni-corrected *p* < 0.0083 (0.05/6) due to the performed tests for six different aortic diameters. Statistical analyses were performed using Stata 16.1 (Stata Corporation).

## Results

The main characteristics of the study sample (*n* = 2668 participants) are provided in Table 1. Participants of the SHIP-START-2 cohort (*N* = 921) were older and had higher average systolic BP compared to the SHIP-TREND-0 sample (*N* = 1747). In addition, all median diameters of the six different thoracic aortic sections were larger in SHIP-START-2 participants than in SHIP-TREND-0 participants.

**Table 1:** Baseline characteristics of the study sample

	Total	SHIP-START-2	SHIP-TREND-0
Parameter	*N* = 2668	*N* = 921	*N* = 1747
Age (years)	53 (43; 63)	56 (45; 65)	52 (41; 62)
Men	1332 (49.9%)	452 (49.1%)	880 (50.4%)
Smoking status			
Never-smoker	1057 (39.6%)	357 (38.8%)	700 (40.1%)
Ex-smoker	1034 (38.8%)	396 (43%)	638 (36.5%)
Current smoker	577 (21.6%)	168 (18.2%)	409 (23.4%)
BMI (kg/m²)	27.1 (24.5; 30.2)	26.9 (24.6; 30.1)	27.2 (24.4; 30.2)
Waist circumference (cm)	89.4 (80; 98.6)	90 (80.3; 98.9)	89 (80; 98.5)
BSA (m^2^)	1.91 (1.76; 2.1)	1.90 (1.75; 2.0)	1.92 (1.77; 2.1)
Systolic blood pressure (mmHg)	128 (116; 139.5)	131 (119.5; 142.5)	126.5 (114.5; 138)
Diastolic blood pressure (mmHg)	78 (71.5; 84.5)	80 (74.0; 86.5)	76.5 (70.5; 83.5)
Diabetes mellitus	228 (8.6%)	86 (9.3%)	142 (8.1%)
HbA1c (%)	5.3 (4.9; 5.6)	5.3 (5;5.7)	5.2 (4.9; 5.6)
HDL-C (mmol/L)	1.41 (1.18; 1.69)	1.40 (1.18; 1.69)	1.41 (1.18; 1.69)
LDL-C (mmol/L)	3.38 (2.77; 3.99)	3.34 (2.73; 3.9)	3.41 (2.81; 4.02)
Total cholesterol (mmol/L)	5.5 (4.8; 6.2)	5.5 (4.7; 6.1)	5.5 (4.8; 6.2)
TGs (mmol/L)	1.36 (0.95; 2.01)	1.54 (1.01; 2.22)	1.29 (0.92; 1.87)
Aortic parameters			
Aortic ascending diameter (cm)	3.36 (3.06; 3.66)	3.40 (3.12; 3.69)	3.33 (3.02; 3.64)
Aortic arch diameter (cm)	2.84 (2.63; 3.08)	2.88 (2.67; 3.12)	2.83 (2.60; 3.06)
Aortic descending diameter (cm)	2.50 (2.25; 2.74)	2.53 (2.30; 2.79)	2.47 (2.24; 2.71)
Aortic subphrenic diameter (cm)	2.34 (2.12; 2.58)	2.39 (2.17; 2.61)	2.32 (2.09; 2.56)
Aortic suprarenal diameter (cm)	2.22 (2.01; 2.41)	2.26 (2.07; 2.44)	2.19 (1.98; 2.39)
Aortic infrarenal diameter (cm)	1.87 (1.72; 2.02)	1.89 (1.74; 2.04)	1.86 (1.70; 2.01)

Data are given as number (percentage) or median (25th and 75th percentile)

*HbA1c* hemoglobin A1c, *HDL-C* high-density lipoprotein cholesterol, *LDL-C* low-density-lipoprotein cholesterol, *BMI* body mass index, *BSA* body surface area, *TG* triglyceride

The overall median of the ascending thoracic aorta was 3.36 cm (IQR: 3.06; 3.66) and of the descending thoracic aorta 2.50 cm (IQR: 2.25; 2.74) (Table 1).

**Fig. 2:**
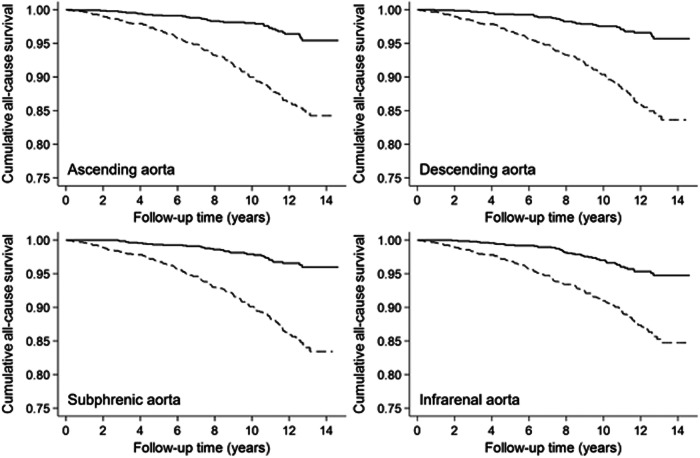
Kaplan–Meier survival estimates for cumulative all-cause mortality for participants with levels above (dashed line) vs below (solid line) the median of selected aortic diameters (log-rank *p* < 0.001, respectively)

**Fig. 3:**
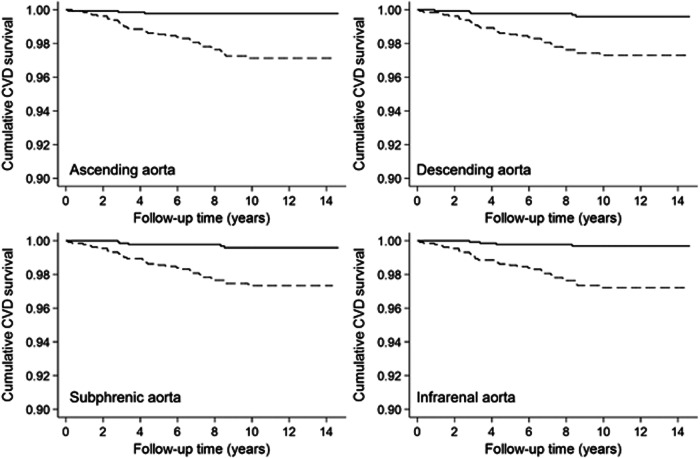
Kaplan–Meier survival estimates for cumulative CVD mortality for participants with levels above (dashed line) vs below (solid line) the median of selected aortic diameters (log-rank *p* < 0.001, respectively)

**Fig. 4:**
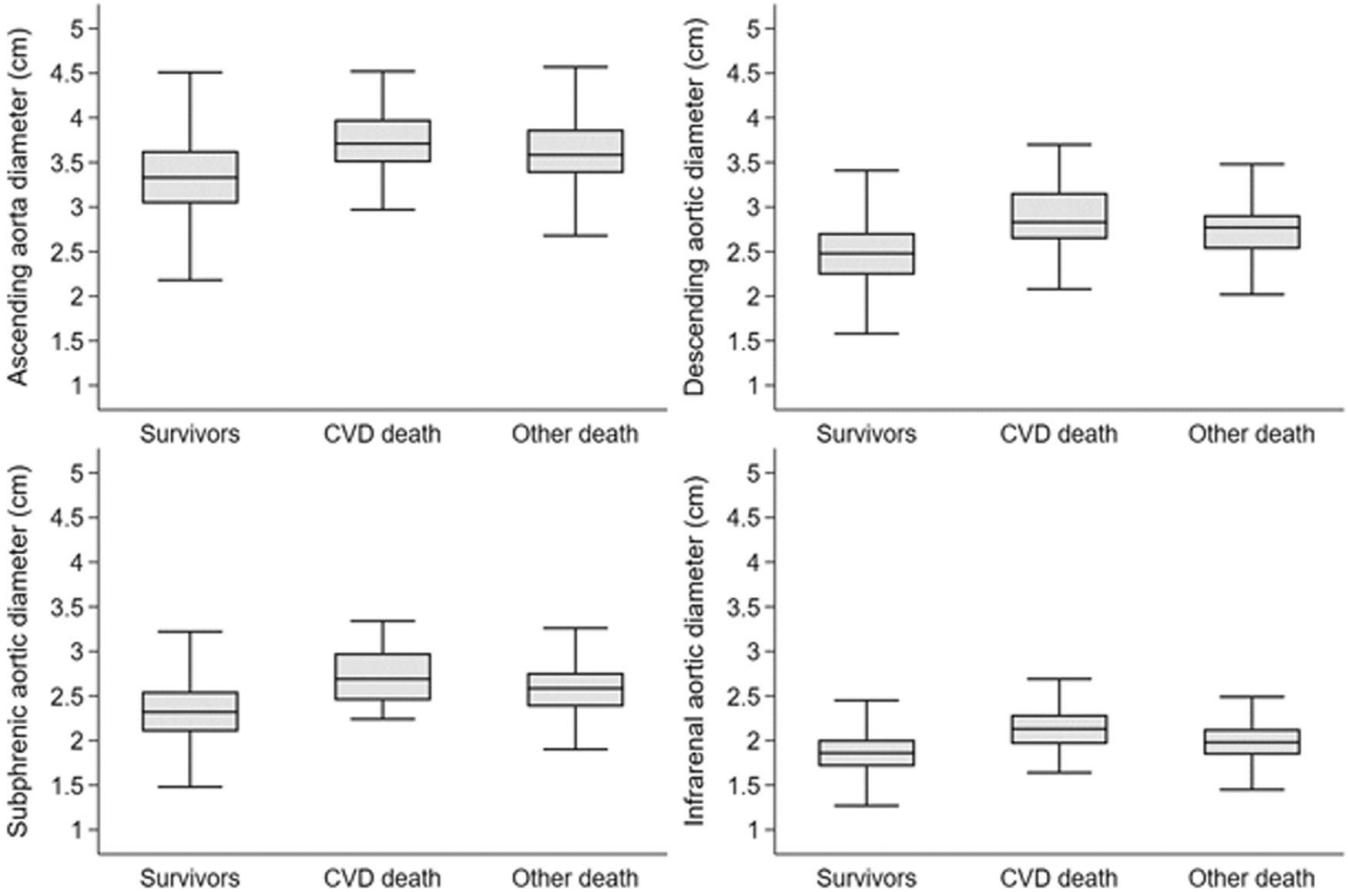
Distribution of aortic diameters displayed by boxplots according to survivors, CVD death, and other deaths after the observed follow-up time

Mortality data were available for a median follow-up time of 10.6 years (IQR: 8.7; 12.4). During 27.702 person-years of follow-up, 188 participants (126 men and 62 women) died, 38 participants (32 men and 6 women) of cardiovascular causes with predominant acute myocardial infarction (*n* = 7, 18%), sudden cardiac death (*n* = 6, 16%), and unspecified heart failure (*n* = 5, 13%). Kaplan–Meier estimates revealed a higher cumulative all-cause mortality in participants with an aortic diameter above the median compared to lower diameters for all investigated aortic sections (all *p* < 0.001, Fig. 2). Similarly, cardiovascular mortality was higher in participants with a higher aortic diameter (all *p* < 0.001; Fig. 3). Aortic diameter distributions according to survivors, CVD death and other death after the observed follow-up time are displayed in Fig. 4.

**Fig. 5:**
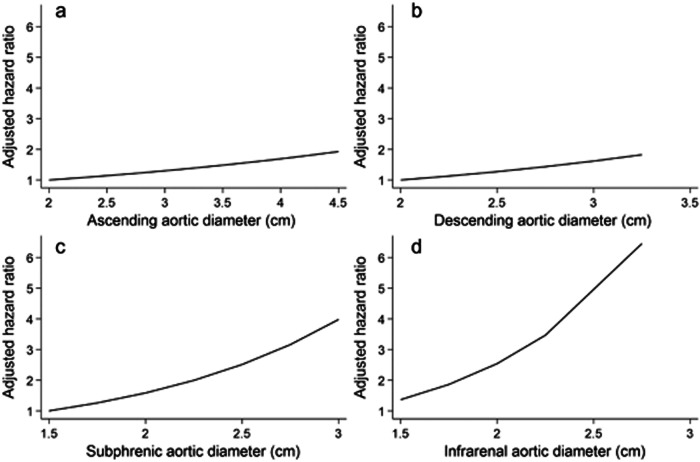
HR of cardiovascular mortality by diameter levels of (**a**) ascending aorta, (**b**) descending aorta, (**c**) subphrenic aorta, and (**d**) infrarenal aorta using multivariable regression splines, adjusted for age and sex, smoking status, BMI, HbA1c, HDL-C, LDL-C, and TG

The ascending thoracic aorta diameter, modeled as a continuous trait, demonstrated an association with cardiovascular mortality (HR = 1.34 95% CI: 1.04; 1.72, *p* = 0.022 per 1-unit increment) but not with all-cause mortality (HR = 1.09 95% CI: 0.83; 1.43, *p* = 0.520) in fully adjusted Cox-proportional hazard regression models. A similar result concerning the association with cardiovascular mortality was found for the infrarenal aorta diameter in the age and sex-adjusted and fully-adjusted model (HR = 3.75 95% CI: 1.06; 13.3, *p* = 0.040; Table 2). The diameter of the subphrenic aorta was associated with higher cardiovascular mortality only in the age and sex-adjusted model (HR = 3.65 95% CI: 1.01; 13.3, *p* = 0.049). Further thoracic aortic section diameters were not associated with mortality of any cause (Table 2). Graphs of multivariable regression spline models displayed linearity of potential associations between thoracic aorta diameters and cardiovascular mortality (Fig. 5). An ascending thoracic aorta diameter above the median was significantly associated with higher cardiovascular mortality (HR = 3.52 95% CI: 1.01; 12.2, *p* = 0.049) and with all-cause mortality (HR = 1.49 95% CI: 1.01; 2.21, *p* = 0.047) in the age and sex-adjusted model but not after multivariable adjustment.

**Table 2:** Association of MRI-derived aortic diameters with cardiovascular and all-cause mortality

Aortic diameter	Cardiovascular mortality		All-cause mortality	
	HR (95% CI)	*p*	HR (95% CI)	*p*
Model 1				
Ascending	1.23 (0.98; 1.54)	0.077	1.06 (0.82; 1.38)	0.657
Arch	1.44 (0.51; 4.03)	0.487	0.94 (0.59; 1.51)	0.807
Descending	1.77 (0.57; 5.50)	0.325	1.12 (0.64; 1.94)	0.688
Subphrenic	3.65 (1.01; 13.25)	0.049	1.41 (0.76; 2.61)	0.281
Suprarenal	2.11 (0.67; 6.65)	0.201	0.81 (0.41; 1.64)	0.565
Infrarenal	4.26 (1.41; 12.91)	0.010	1.13 (0.56; 2.29)	0.734
Model 2				
Ascending	1.34 (1.04; 1.72)	0.022	1.09 (0.83; 1.43)	0.520
Arch	1.48 (0.47; 4.69)	0.504	1.00 (0.60; 1.65)	0.992
Descending	1.50 (0.40; 5.66)	0.548	0.93 (0.51; 1.71)	0.823
Subphrenic	2.46 (0.59; 10.3)	0.217	1.08 (0.56; 2.08)	0.814
Suprarenal	1.57 (0.38; 6.47)	0.531	0.63 (0.31; 1.31)	0.218
Infrarenal	3.75 (1.06; 13.3)	0.040	0.93 (0.43; 2.01)	0.858

HR per 1-unit increment in aortic diameter from Cox-proportional hazard regression models adjusted for age and sex (Model 1) and additionally for smoking status, BMI, HbA1c, HDL-C, LDL-C, and TG (Model 2)

Effect modification analysis revealed a stronger impact of aorta diameters on cardiovascular mortality in participants with a higher BMI above the median (ascending thoracic aorta HR = 5.35 95% CI: 2.06; 13.9, *p* = 0.001) (Table 3).

**Table 3:** Association of MRI-derived aortic diameters with cardiovascular and all-cause mortality according to BMI group

	Cardiovascular mortality		All-cause mortality	
Aortic diameter	HR (95% CI)	*p*	HR (95% CI)	*p*
BMI > median				
Ascending	5.35 (2.06; 13.9)	0.001	1.34 (0.83; 2.15)	0.227
Arch	8.26 (2.17; 31.4)	0.002	1.29 (0.7; 2.38)	0.417
Descending	9.89 (2.56; 38.2)	0.001	1.39 (0.71; 2.72)	0.337
Subphrenic	22.1 (3.97; 123.2)	< 0.001	1.09 (0.50; 2.35)	0.831
Suprarenal	4.53 (1.89; 10.8)	0.001	0.89 (0.41; 1.95)	0.774
Infrarenal	13.7 (3.45; 54.6)	< 0.001	1.28 (0.56; 2.95)	0.557
BMI ≤ median				
Ascending	1.08 (0.38; 3.09)	0.889	1.13 (0.85; 1.51)	0.410
Arch	0.33 (0.05; 2.04)	0.234	1.30 (0.59; 2.84)	0.519
Descending	0.37 (0.05; 2.74)	0.333	1.28 (0.50; 3.23)	0.607
Subphrenic	1.03 (0.14; 7.54)	0.978	2.85 (1.11; 7.30)	0.029
Suprarenal	0.74 (0.08; 7.38)	0.801	1.60 (0.56; 4.55)	0.382
Infrarenal	3.30 (0.44; 24.6)	0.244	2.06 (0.71; 5.91)	0.181

HR per 1-unit increment in aortic diameter from Cox-proportional hazard regression models adjusted for age, sex, smoking status, BMI, HbA1c, HDL-C, LDL-C, and TG

Model discrimination was improved slightly by adding the median-dichotomized ascending thoracic aorta diameter to a standard risk factor model for all-cause mortality (C-statistic, 0.8254 vs 0.8220, *p* = 0.019) and for cardiovascular mortality (C-statistic, 0.8854 vs 0.8732, *p* = 0.049).

In sensitivity analysis, a number of five deaths that occurred during the first year of follow-up were excluded without substantial change in the association between the ascending thoracic aortic diameter (HR = 1.34 95% CI: 1.05; 1.70, *p* = 0.019) and the infrarenal aortic diameter (HR = 4.45 95% CI: 1.28; 15.5, *p* = 0.019) with higher cardiovascular mortality.

Continuous aortic MRI parameters showed no significant associations with cardiovascular and all-cause mortality when indexed by BSA (Table 4 and BMI (Table 5).

**Table 4:** Association of MRI-derived aortic diameters (BSA-indexed) with cardiovascular and all-cause mortality

	Cardiovascular mortality		All-cause mortality	
Aortic diameter/BSA	HR (95% CI)	*p*	HR (95% CI)	*p*
Model 1				
Ascending	1.28 (0.71; 2.29)	0.414	0.85 (0.42; 1.72)	0.654
Arch	0.57 (0.07; 4.75)	0.601	0.53 (0.21; 1.34)	0.180
Descending	0.86 (0.07; 10.3)	0.907	0.67 (0.22; 2.04)	0.481
Subphrenic	2.48 (0.20; 31.1)	0.481	0.92 (0.29; 2.93)	0.888
Suprarenal	0.96 (0.06; 15.3)	0.974	0.34 (0.10; 1.20)	0.095
Infrarenal	7.46 (0.56; 99.9)	0.129	0.59 (0.14; 2.42)	0.463
Model 2				
Ascending	1.48 (0.86; 2.55)	0.156	1.03 (0.56; 1.91)	0.921
Arch	0.83 (0.08; 8.12)	0.871	0.75 (0.29; 1.97)	0.563
Descending	1.17 (0.08; 16.8)	0.907	0.68 (0.21; 2.19)	0.523
Subphrenic	1.95 (0.14; 27.9)	0.624	0.80 (0.24; 2.65)	0.719
Suprarenal	0.80 (0.05; 13.3)	0.878	0.32 (0.09; 1.14)	0.079
Infrarenal	6.56 (0.46; 92.6)	0.164	0.57 (0.13; 2.46)	0.448

HR per 1-unit increment in aortic diameter from Cox-proportional hazard regression models adjusted for age and sex (Model 1) and additionally for smoking status, HbA1c, HDL-C, LDL-C, and TG (Model 2)

**Table 5:** Association of MRI-derived aortic diameters (BMI-indexed) with cardiovascular and all-cause mortality

	Cardiovascular mortality		All-cause mortality	
Aortic diameter/BSA	HR (95% CI)	*p*	HR (95% CI)	*p*
Model 1				
Ascending	1.02 (0.70; 1.49)	0.904	0.95 (0.79; 1.15)	0.602
Arch	0.86 (0.61; 1.22)	0.393	0.92 (0.79; 1.07)	0.268
Descending	0.86 (0.60; 1.24)	0.421	0.94 (0.81; 1.1)	0.466
Subphrenic	0.97 (0.68; 1.39)	0.879	0.98 (0.84; 1.15)	0.826
Suprarenal	0.90 (0.63; 1.29)	0.567	0.91 (0.78; 1.06)	0.226
Infrarenal	1.11 (0.80; 1.54)	0.516	0.96 (0.83; 1.11)	0.588
Model 2				
Ascending	1.13 (0.85; 1.50)	0.408	1.02 (0.86; 1.22)	0.794
Arch	0.94 (0.64; 1.39)	0.767	0.98 (0.84; 1.16)	0.853
Descending	0.93 (0.62; 1.39)	0.728	0.97 (0.82; 1.15)	0.765
Subphrenic	1.00 (0.68; 1.49)	0.991	1.00 (0.85; 1.19)	0.981
Suprarenal	0.93 (0.63; 1.37)	0.715	0.92 (0.78; 1.09)	0.343
Infrarenal	1.16 (0.84; 1.62)	0.363	0.98 (0.84; 1.16)	0.848

HR per 1-SD increment in aortic diameter from Cox-proportional hazard regression models adjusted for age and sex (Model 1) and additionally for smoking status, HbA1c, HDL-C, LDL-C, and TG (Model 2)

## Discussion

In this population-based analysis including 2668 participants (1336 women) free of known CVD, we assessed the associations of 6 different segments of the thoracic and abdominal aorta with all-cause and cardiovascular mortality. We observed that the non-indexed diameters of the ascending thoracic aorta and of the infrarenal aorta were statistically significantly associated with higher cardiovascular mortality in multivariable-adjusted models, but not with all-cause mortality.

The associations of aortic diameters with risk factors of CVD events and mortality were examined in several studies with partially conflicting results (3–6). In two cohorts of the Framingham Heart Study (mean follow-up 8.8 years, non-contrast computed tomography (CT) of the thoracic and abdominal aorta) increased diameter of the infrarenal and of the lower abdominal aorta conferred a 57%, respectively 53% greater risk for incident CVD events (including cardiovascular death) [6].

In the Rotterdam study (mean follow-up 9 years), an association between BMI-indexed ascending (33% higher risk) and descending thoracic aortic diameter (38% higher risk) and cardiovascular mortality for women, as well as all-cause mortality for women and men, was reported [5]. However, there was no association with mortality for non-indexed aortic diameters.

Our results partly support the observations of both studies, mentioned above. Consistent with the observations in the Framingham cohorts, the diameter of the infrarenal aorta was associated with increased CVD mortality in our sample. In Framingham, the association with a combined endpoint, consistent with CVD death, myocardial infarction, coronary insufficiency, index admission for heart failure, and stroke was investigated [6].

We also observed an association of the ascending aorta diameter with CVD mortality, as in the Rotterdam study. However, in contrast to the finding of the Rotterdam Study, we observed these associations only for the non-indexed aortic diameters, but not for BMI- or BSA-indexed diameters. However, the most appropriate method for normalizing aortic diameter measurements is still debated. While indexation for BSA is often used in the literature [13]. There is some evidence, that in underweight and overweight subjects, correction for BSA can distort the prevalence of left ventricular hypertrophy [14]. It is conceivable that this also applies to the aortic diameter measurements [15]. A reason for our findings could be a higher proportion of overweight participants in our cohort compared to the participants of the Rotterdam Study, with a subsequent underestimation of the indexed aortic diameter in obese patients.

The ascending aorta plays a central role in cushioning the highly pulsatile blood flow from the left ventricle into the steady, non-pulsatile flow, which is delivered to the target organs. Arterial aging throughout the lifespan is associated with a decrease of this function resulting in subclinical damage to high-flow, low-resistance organs such as the heart, brain, and kidney increasing the risk for cardiovascular mortality [8, 16].

The infrarenal part of the aorta and its association with cardiovascular mortality is interesting from a practical view, because of its easy approach using ultrasound and the fact that this aortic segment is prone to dilate, which makes it a common location for aneurysm formation. Ultrasound-based studies could confirm the association of increased infrarenal aortic diameter with future CVD events, as well as cardiovascular and all-cause mortality [17, 18].

However, our study does not support all the results of the above-mentioned studies. Differences in the samples (e.g., with respect to the distribution of age or of standard CVD risk factors), in the exact endpoints used (incident CVD as defined above, cardiovascular mortality, all-cause mortality), indexing of the exposure variables (the aortic diameters to BMI or BSA) and different imaging modalities (ultrasound vs CT vs MRI) might contribute to the observed differences.

In addition, we observed that in participants with a higher BMI (above the median), a stronger association between the ascending thoracic aorta on cardiovascular mortality was present, whereas in participants with a lower BMI (below the median), a stronger association of the ascending thoracic aorta with all-cause mortality was observed.

The reasons for the association of aortic diameter with all-cause mortality are not well understood. However, age-associated vascular remodeling including an increase in diameter activates a multifactorial, inflammatory response. This may explain the association of increased aortic diameters with nonvascular causes of death including malignancy, infection, and neurodegenerative disorders, that are also characterized by a chronic inflammatory state [8, 19, 20].

To our knowledge, this is the first study assessing the associations of cardiovascular and all-cause mortality risks with thoracic and abdominal aortic diameters in a population-based approach. A limitation of our study is the relatively small number of deaths, which could be observed during the follow-up period. However, several sensitivity analyses could confirm our main finding, even after considering the Bonferroni-adjusted level of significance. In addition, we had no information about connective tissue or aortic valve disease of the participants, both is associated especially with the diameter of the ascending aorta and we did not analyze the association of aortic diameter with CVD events (e.g., myocardial infarction or stroke) as other studies did previously. Finally, we used only plain diameter measurements, whereas the determination of the cross-sectional vascular area would produce more valid results.

In conclusion, the results of this study indicate that greater non-indexed diameters of the ascending thoracic aorta and of the infrarenal aorta are associated with higher cardiovascular mortality. Physicians analyzing cross-sectional images should be aware of this association. Future research is needed to answer the question if there is a prognostic role of aortic diameter measurement to predict cardiovascular mortality and to evaluate how they can be included in an improved prevention process.

## Abbreviations

BMI: Body mass index
BSA: Body surface area
CI: Confidence interval
CVD: Cardiovascular disease
HbA1c: Glycated hemoglobin
HDL-C: High-density lipoprotein cholesterol
HR: Hazard ratios
IQR: Interquartile range
LDL-C: Low-densitiy lipoprotein cholesterol
SHIP: Study of Health in Pomerania
TG: Triglyceride
